# Comparative significance of piperacillin-tazobactam and cefotaxime-tazobactam in the treatment of respiratory tract infections in the elderly

**DOI:** 10.3389/fphar.2026.1757937

**Published:** 2026-08-03

**Authors:** Mengzhe Peng, Henian Liang, Jinwen Chen, Junli Zhang, Dongyun Qin

**Affiliations:** 1 School of Pharmacy, Guangdong Medical University, Zhanjiang, Guangdong, China; 2 Department of Pharmacy, Zhongshan City People’s Hospital, Zhongshan, Guangdong, China

**Keywords:** cefotaxime-tazobactam, comparative efficacy, elderly, lower respiratory tract infection, piperacillin-tazobactam

## Abstract

**Objective:**

To compare the clinical outcomes associated with piperacillin-tazobactam (PIP-TAZ) versus cefotaxime-tazobactam (CTX-TAZ) in elderly patients with lower respiratory tract infection (LRTI).

**Methods:**

A total of 92 elderly patients with respiratory tract infection from March 2024 to April 2025 were selected as the subjects and divided into two groups of 46 cases in each group according to different treatment drugs. The control group was treated with tazobactam sodium/cefotaxime sodium, and the observation group was treated with piperacillin sodium for injection. The effects of the two groups were evaluated after 2 weeks of treatment. The blood gas level, inflammatory factors, lung function and drug safety were compared between the two groups.

**Results:**

Both groups showed significant improvements in arterial blood gases after treatment. Patients receiving PIP-TAZ exhibited a significantly greater increase in PaO2 and a greater reduction in PaCO2 compared to those receiving CTX-TAZ (both *P* < 0.05). Inflammatory markers (PCT, WBC, CRP) decreased, and pulmonary function indices (PEF, FVC, FEV1) increased significantly in both groups, with numerically greater improvements in the PIP-TAZ group (all *P* < 0.05, unadjusted). There was no statistical difference in drug safety between the two groups (*P* > 0.05).

**Conclusion:**

In this comparative study, both piperacillin-tazobactam and cefotaxime-tazobactam demonstrated clinical effectiveness in improving objective physiological and inflammatory parameters in elderly patients with LRTIs. In this non-randomized comparison, the piperacillin-tazobactam regimen was associated with statistically significant, but unadjusted, differences favoring greater improvements in arterial blood gases (PaO2 and PaCO2), reductions in inflammatory biomarkers (PCT, WBC, CRP), and enhancements in lung function indices (PEF, FVC, FEV1) after 2 weeks of treatment compared to the cefotaxime-tazobactam regimen. These observed differences should be considered exploratory. Both treatments exhibited a comparable and acceptable safety profile in this cohort. However, these findings should be interpreted in light of the study’s non-randomized design, heterogeneous infection types, and the inherent differences between the antibiotic regimens. Further large-scale, randomized controlled trials with microbiological confirmation are warranted to validate these observations and define the optimal empiric antibiotic strategy in this vulnerable population.

## 1 Introduction

The pathogenesis of respiratory tract infections in the elderly is complex. As patients age, their lung function declines, and their local and systemic immunity decreases. Aging occurs in all aspects of cellular immunity, humoral immunity, innate immunity, and acquired immunity (Ji et al., 2024). Some elderly patients are accompanied by chronic degenerative diseases, such as cardiovascular and cerebrovascular diseases and chronic renal failure, and environmental factors increase the incidence of respiratory tract infections (Wang and Zhou, 2024). Cefoperazone sodium belongs to the third-generation cephalosporin antibiotics, which exerts its antibacterial effect by inhibiting bacterial cell wall synthesis. The addition of tazobactam sodium expands the antibacterial spectrum of cefoperazone sodium. The combination of the two drugs can exert a systemic antibacterial effect and enhance the overall antibacterial effect (Chen et al., 2023). Piperacillin sodium for injection is a β-lactam antibiotic that exerts its antibacterial effect by inhibiting bacterial cell wall synthesis. Piperacillin sodium has good antibacterial effects on a variety of Gram-positive and Gram-negative bacteria and has a wide range of application value (Mauritz et al., 2024). At the same time, piperacillin sodium for injection has good stability in the patient’s body, is quickly absorbed and distributed to various tissues and organs, and exerts an antibacterial effect. These pharmacological properties suggest a potential to alleviate infection-related symptoms and support patient recovery (Wang, M. L. et al., 2023). This study aims to compare the efficacy of piperacillin sodium for injection and tazobactam sodium/cefotaxime sodium in elderly patients with respiratory tract infections.

## 2 Subjects and methods

### 2.1 Study design and participants

This was a single-center, prospective, non-randomized comparative study conducted between March 2024 and April 2025. A total of 92 elderly patients (aged ≥65 years) diagnosed with lower respiratory tract infection (LRTI) were enrolled. Sample size calculation was performed *a priori* based on a two-sample comparison of means for the primary outcome of PaO2 improvement, assuming a mean difference of 5 mmHg, standard deviation of 6 mmHg, 80% power, and a two-sided alpha of 0.05, which yielded a required sample size of 44 patients per group. Considering potential dropouts, 46 patients per group were included. Patients were allocated to one of two groups according to a sequential assignment scheme based on admission date (odd vs. even days), combined with clinical judgment and drug availability, aiming to achieve comparable baseline characteristics. The PIP-TAZ group (n = 46) received piperacillin-tazobactam, and the CTX-TAZ group (n = 46) received cefotaxime-tazobactam. Assessors of laboratory and pulmonary function outcomes were blinded to group allocation. No significant differences were observed in baseline characteristics between the two groups (*P* > 0.05), as shown in Tables 1, 2.

**TABLE 1:** Comparison of general information between the two groups.

Group	Number of cases	Gender (male/female)	Body mass index (kg/m^2)^	Age (years)	Disease duration (d)	Comorbidities (yes/no)
Observation group	46	28/18	22.46 ± 3.41	69.88 ± 5.73	6.78 ± 1.12	15/31
Control group	46	25/21	22.49 ± 3.45	71.13 ± 5.78	6.82 ± 1.15	13/33
*χ* ^*2*^ */t*	—	0.401	0.042	1.042	0.169	0.205
*P*	—	0.527	0.967	0.300	0.866	0.650

**TABLE 2:** Comparison of additional baseline clinical management characteristics between the two groups [n (%)].

Characteristic	Observation group (n = 46)	Control group (n = 46)	χ^2^/t	P
Supplemental oxygen	34 (73.9)	32 (69.6)	0.213	0.645
Non-invasive ventilation	8 (17.4)	7 (15.2)	0.082	0.775
Bronchodilators	22 (47.8)	20 (43.5)	0.176	0.675
Theophylline-based drugs	12 (26.1)	11 (23.9)	0.059	0.808
Nebulizer inhalation therapy	18 (39.1)	16 (34.8)	0.188	0.665

No significant differences were observed between the two groups in any of the listed clinical management characteristics (all *P* > 0.05).

### 2.2 Inclusion and exclusion criteria

Inclusion criteria: (1) Patients aged ≥65 years who met the diagnostic criteria for lower respiratory tract infection (LRTI), including community-acquired pneumonia (CAP) and acute exacerbations of chronic obstructive pulmonary disease (AECOPD) or chronic bronchitis, as per relevant guidelines (Expert Committee of Chinese Laboratory Medicine Training Project Chinese Society of Respiratory Medicine et al., 2023) and clinical assessment. (2) Patients diagnosed based on clinical symptoms (e.g., cough, sputum, dyspnea, fever), chest radiograph or computed tomography findings consistent with infection, and elevated inflammatory markers (e.g., CRP, PCT, WBC). (3) Patients with no history of allergy or contraindications to tazobactam sodium/cefotaxime sodium and piperacillin sodium for injection. Exclusion criteria: (1) Patients with mental disorders or other respiratory diseases. (2) Patients who have used antibiotics in the past 2 weeks or have infections in other parts of the body. (3) Patients with severe liver and kidney dysfunction and confirmed malignant tumors.

### 2.3 Methods

Both groups received conventional supportive treatment, including supplemental oxygen administered via nasal cannula or Venturi mask with titrated FiO_2_ to maintain oxygen saturation ≥90%, as clinically indicated. In cases of respiratory failure, non-invasive positive pressure ventilation was initiated when clinically warranted. Hydration, and symptomatic management (e.g., antipyretics, antitussives, bronchodilators as clinically indicated) were also provided. Etiological testing, including sputum culture and/or blood culture, was attempted for all patients at enrollment but was not universally feasible or conclusive. The rate of positive microbiological confirmation and the distribution of identified pathogens are not reported herein, as the study was not powered for pathogen-specific analysis. Empirical antibiotic treatment was initiated promptly based on clinical judgment. For patients with fever, antipyretic drugs such as acetaminophen or ibuprofen can be given. For patients with cough, expectorant and antitussive drugs such as ambroxol and dextromethorphan can be given. For those with nasal congestion and runny nose, drugs such as pseudoxanthine can be used to relieve symptoms. For those with shortness of breath, chest tightness and asthma symptoms, theophylline drugs or nebulizer inhalation treatment can be given appropriately (Xiao and Liu, 2025).

Control group was treated with tazobactam sodium/cefotaxime sodium. Take tazobactam sodium/cefotaxime sodium injection (Hainan Meihaoxilin Biopharmaceutical Co., Ltd., national medicine standard H20183493, specification: 2.0 g (C25H27N9O8S2 1.6 g and C10H12N4O5S 0.4 g)) 3.0 g mixed in 100 mL normal saline, mixed evenly and then intravenously dripped, the drip time should not be less than 30 min, 3 times a day, and continuous treatment for 2 weeks (1 course of treatment). Piperacillin-tazobactam (PIP-TAZ) group: Patients received piperacillin-tazobactam (Zhejiang Jinhua Kangenbei Biopharmaceutical Co., Ltd., National Drug Approval No. H20153285, specification: 1.125 g per vial containing piperacillin 1.00 g and tazobactam 0.125 g) at a dose of 4.5 g (4 g piperacillin/0.5 g tazobactam) every 8 h, reconstituted in 100 mL of normal saline, administered by intravenous infusion over 30 min, for a total duration of 2 weeks. It is acknowledged that the two regimens differ in their antibiotic composition (a third-generation cephalosporin combined with a β-lactamase inhibitor versus an extended-spectrum penicillin combined with a β-lactamase inhibitor), dosing frequency, and pharmacokinetic properties, which are important contextual factors for the observed outcomes.

### 2.4 Observation indicators

(1) Blood gas levels. Blood gas analyzers were used to measure oxygen partial pressure (PaO2), carbon dioxide partial pressure (PaCO2) and pH levels in the two groups before and after the intervention (Zhang et al., 2024). (2) Inflammatory factors and lung function. Automatic biochemical analyzers were used to measure procalcitonin (PCT) and white blood cell count (WBC) levels in the two groups before and after the intervention (Chai et al., 2024). C-reactive protein (CRP) levels were measured by immunoturbidimetry. Pulmonary function tester was used to measure PEF, FVC and FEV1 levels (Shi, 2024). (3) Drug safety. The incidence of nausea and vomiting, phlebitis, edema, hypotension, diarrhea and constipation in the two groups was statistically analyzed.

### 2.5 Statistic analysis

SPSS 28.0 software (IBM Corp., Armonk, NY, USA) was used for analysis. Categorical variables (e.g., gender, comorbidities, adverse event incidence) were presented as number (percentage) and compared using the Chi-square test or Fisher’s exact test as appropriate. Continuous variables (e.g., age, laboratory parameters, lung function indices) were tested for normality using the Shapiro-Wilk test. Normally distributed data were expressed as mean ± standard deviation (SD) and compared between groups using the independent samples t-test. Within-group comparisons before and after treatment were performed using the paired samples t-test. A two-tailed *P* value < 0.05 was considered statistically significant. No adjustment for multiple comparisons was performed given the exploratory nature of these secondary endpoint analyses; therefore, findings regarding individual inflammatory markers and lung function parameters should be interpreted with extreme caution, acknowledging an increased risk of type I error. All statistical inferences, particularly for the numerous secondary endpoints, are considered exploratory and hypothesis-generating rather than confirmatory.

## 3 Results

### 3.1 Baseline respiratory support and oxygen therapy

At admission, 34 patients (73.9%) in the observation group and 32 patients (69.6%) in the control group received supplemental oxygen via nasal cannula or Venturi mask. Non-invasive positive pressure ventilation was initiated in 8 patients (17.4%) in the observation group and 7 patients (15.2%) in the control group. No patient in either group required invasive mechanical ventilation at baseline. The distribution of respiratory support modalities was comparable between the two groups (*P* > 0.05 for all comparisons).

### 3.2 Comparison of blood gas levels between the two groups

The blood gas levels of the two groups improved after intervention. There was no statistical difference in pH value between the two groups before and after intervention (*P* > 0.05). The PaO2 in the observation group was higher than that in the control group (*P* < 0.05). The PaCO2 was lower than that in the control group (*P* < 0.05), see Table 3. It is important to note that these between-group comparisons, like all others in this study, are unadjusted for multiple testing and should be interpreted in the context of the study’s exploratory design.

**TABLE 3:** Comparison of blood gas levels between the two groups ( $\bar{x}\pm s$ ).

Group	Number of cases	PaO2 (mmHg)		pH		PaCO2 (mmHg)	
		Before intervention	After intervention	Before intervention	After intervention	Before intervention	After intervention
Observation group	46	57.91 ± 4.53	73.25 ± 6.48^a^	7.12 ± 0.51	7.23 ± 0.57	55.29 ± 4.51	42.84 ± 3.26^a^
Control group	46	58.11 ± 4.56	61.49 ± 5.63^a^	7.15 ± 0.54	7.18 ± 0.55	55.34 ± 4.55	48.91 ± 4.12^a^
*t*	—	0.211	9.292	0.274	0.428	0.053	7.836
*P*	—	0.833	0.000	0.785	0.670	0.958	0.000

Compared with the group before intervention.

^a^ *P* < 0.05. Despite significant improvements in PaCO_2_, post-treatment pH values remained within the mildly acidic range in both groups, suggesting the presence of concomitant metabolic acidosis, which may be attributable to underlying comorbidities, subclinical renal functional impairment, or the systemic inflammatory response despite the exclusion of severe renal dysfunction.

### 3.3 Comparison of inflammatory factors and lung function between the two groups

After intervention, the inflammatory response in the two groups was alleviated and the lung function was improved. The PCT, WBC and CRP in the observation group were lower than those in the control group (*P* < 0.05). The PEF, FVC and FEV1 levels were higher than those in the control group (*P* < 0.05), as shown in Table 4. The statistical significance of differences across the multiple inflammatory and pulmonary function indices evaluated must be viewed cautiously due to the lack of multiplicity adjustment.

**TABLE 4:** Comparison of inflammatory factors and lung function between the two groups ( $\bar{x}\pm s$ ).

Group	Number of cases	PCT (μg/L)		WBC (×10^9^/L)		CRP (mg/L)	
		Before intervention	After intervention	Before intervention	After intervention	Before intervention	After intervention
Observation group	46	3.51 ± 0.45	0.63 ± 0.11^a^	17.84 ± 2.31	8.46 ± 1.04^a^	52.59 ± 4.51	10.26 ± 1.36^a^
Control group	46	3.54 ± 0.49	1.58 ± 0.32^a^	17.87 ± 2.36	12.51 ± 1.59^a^	52.62 ± 4.55	19.68 ± 2.52^a^
*T value*	—	0.301	19.452	0.062	15.671	0.032	24.836
*P value*	—	0.764	<0.001	0.951	<0.001	0.975	<0.001

Group	Number of cases	PEF (L/s)		FVC (L)		PEF1 (L)	
		Before intervention	After intervention	Before intervention	After intervention	Before intervention	After intervention
Observation group	46	1.77 ± 0.45	2.97 ± 0.62^a^	1.81 ± 0.45	3.22 ± 0.58^a^	1.34 ± 0.26	2.43 ± 0.32^a^
Control group	46	1.79 ± 0.48	2.15 ± 0.51^a^	1.78 ± 0.42	2.35 ± 0.51^a^	1.31 ± 0.24	2.01 ± 0.28^a^
*T value*	—	0.206	6.928	0.331	7.640	0.575	6.699
*P value*	—	0.837	<0.001	0.742	<0.001	0.567	<0.001

Compared with the group before intervention

^a^ *P* < 0.05.

### 3.4 Comparison of drug safety between the two groups

There was no statistical difference in drug safety between the two groups (P > 0.05), see Table 5.

**TABLE 5:** Comparison of drug safety between the two groups [n (%)].

Group	Number of cases	Nausea and vomiting	Phlebitis	Edema	Low blood pressure	Diarrhea and constipation	Incidence
Observation group	46	2 (4.35)	0 (0.00)	1 (2.17)	0 (0.00)	1 (2.17)	4 (8.70)
Control group	46	1 (2.17)	1 (2.17)	1 (2.17)	1 (2.17)	1 (2.17)	5 (10.87)
$x^{2}$	—	—	—	—	—	—	0.123
*P*	—	—	—	—	—	—	0.726

## 4 Discussion

Elderly patients with respiratory tract infections have a further decrease in PaO_2_ levels due to decreased lung function, ventilation/perfusion imbalance, or increased intrapulmonary shunt. With continued decrease in PaO_2_, the patient’s oxygenated hemoglobin dissociation curve enters a steep section, resulting in a sharp drop in the patient’s blood oxygen saturation, indicating impaired respiratory function (Wang, X. 2023). In this study, blood gas levels in both groups improved after intervention. The PaO_2_ in the observation group was higher than that in the control group (*P* < 0.05). PaCO_2_ was lower than that in the control group (*P* < 0.05). From this result, both regimens were associated with improved blood gas levels of elderly patients with respiratory tract infections and facilitated their recovery, and the observed differences favored the PIP-TAZ group. Speculatively, these observed differences might be explained by factors including the broad antibacterial spectrum, rapid onset, and strong penetration of piperacillin, but such mechanistic interpretations remain hypothetical and should not be construed as evidence of comparative effectiveness. While piperacillin possesses pharmacological properties such as activity against a variety of Gram-negative bacteria and good tissue penetration (Liu et al., 2025a; Milani et al., 2024), these inherent drug characteristics cannot, in themselves, account for the observed between-group differences. This is because the study’s non-randomized allocation and the lack of microbiological confirmation of pathogens and their susceptibilities preclude any causal attribution of clinical outcomes to these specific pharmacological attributes.

PCT, as a common inflammatory indicator, can guide anti-infection treatment for elderly patients with respiratory tract infections. When this indicator increases, it indicates a high possibility of bacterial infection and it is recommended to use antibiotics. Therefore, dynamic monitoring of PCT levels in clinical practice can evaluate the treatment effect of patients and guide the use of antibiotics. WBC, as a cell type in human blood, is mainly responsible for the body’s immune defense function. When the body is infected by bacteria, viruses and other factors, white blood cells can deform and pass through the capillary wall, concentrate at the site of bacterial invasion, surround and phagocytize the bacteria, and cause an increase in WBC levels (Zhao and Ou, 2024). CRP is an acute phase protein produced by the liver and is expressed at a low level in normal humans. When the body is infected with bacteria, it will cause a sharp increase in CRP levels and reach a peak in a short period of time. Its expression level is related to the degree of infection or tissue damage in the patient. In this study, the inflammatory response in the body was alleviated and lung function was improved after intervention in both groups. PCT, WBC and CRP in the observation group were lower than those in the control group (*P* < 0.05). PEF, FVC and FEV1 levels were higher than those in the control group (*P* < 0.05). There was no statistical difference in the safety of the two groups of drugs (*P* > 0.05). In this exploratory analysis, both regimens were associated with reductions in inflammatory factors in elderly patients with respiratory tract infections, helped improve patients’ lung function, and did not significantly increase the incidence of adverse drug reactions. The numerical differences favored the PIP-TAZ group. However, as previously emphasized, these findings are exploratory and do not imply causal superiority. Alternative explanations related to study design, clinical heterogeneity, and unmeasured confounders cannot be ruled out. Therefore, any clinical recommendations based on these results would be premature (Liu et al., 2025b). Future research should aim to validate these observations in rigorously designed randomized controlled trials with microbiological confirmation. In this study, the majority of patients in both groups received supplemental oxygen via conventional devices, with a small proportion requiring non-invasive ventilation. The comparable distribution of respiratory support modalities between groups strengthens the validity of the observed differences in blood gas improvements, as the baseline severity of hypoxemia and the level of respiratory support were well balanced. These findings suggest that the greater improvement in PaO_2_ and PaCO_2_ observed in the piperacillin-tazobactam group cannot be solely attributed to differences in oxygen delivery strategies.

Recent international guidelines and studies also highlight the importance of appropriate empiric antibiotic selection in elderly patients with LRTIs, considering local resistance patterns and patient comorbidities (Metlay et al., 2019; Grau et al., 2014). However, the present study’s design does not permit conclusions regarding which regimen is more effective; rather, it underscores the need for future comparative effectiveness research in this population.

Although patients with severe renal dysfunction were excluded, the persistently mildly acidic post-treatment pH values observed in both groups suggest the presence of concurrent metabolic acidosis. This phenomenon may be explained by several factors commonly present in elderly patients with lower respiratory tract infections, including age-related decline in renal functional reserve, the impact of systemic inflammation on acid-base balance, and potential subclinical lactic acidosis secondary to hypoxemia or tissue hypoperfusion. These findings highlight the complexity of acid-base disturbances in the elderly population and underscore the importance of comprehensive metabolic monitoring beyond respiratory parameters.

Several important differences between the treatment regimens warrant consideration. The PIP-TAZ group received therapy every 8 h, while the CTX-TAZ group received it three times daily (approximate 8-h intervals as well), aligning with their respective pharmacokinetic/pharmacodynamic (PK/PD) principles for time-dependent antibiotics. The similar dosing frequencies likely mitigated potential bias due to PK/PD differences, though the extended-spectrum penicillin (piperacillin) and third-generation cephalosporin (cefotaxime) have distinct spectra and tissue penetration properties, which might hypothetically explain the observed efficacy differences, though this remains speculative. However, the non-randomized allocation scheme (based on admission date and clinical judgment) remains a significant limitation, as it cannot fully eliminate the risk of selection and indication bias despite statistically comparable baseline characteristics. Unmeasured confounders related to disease severity, comorbidities, or pathogen distribution may have influenced treatment assignment and outcomes. The strengths of this study include the use of objective and multi-dimensional outcome measures, such as arterial blood gas analysis, specific inflammatory biomarkers (PCT, CRP, WBC), and standardized pulmonary function tests, which provide a comprehensive assessment of treatment response beyond clinical symptoms alone.

Furthermore, the clinical heterogeneity of the enrolled LRTI subtypes (encompassing CAP, AECOPD, and chronic bronchitis) represents an important confounding factor. The observed differences in treatment response could be influenced by the underlying infection type rather than, or in addition to, the antibiotic regimen itself. This further limits the ability to draw causal conclusions regarding comparative effectiveness. Future studies should aim for more homogeneous patient populations or incorporate pre-specified subgroup analyses to better isolate treatment effects.

## 5 Limitations

This study has several limitations that should be considered when interpreting the results. First, the prospective but non-randomized, open-label design with allocation based on admission date and clinician discretion carries a substantial risk of selection and indication bias. This methodological constraint severely limits causal inference and the strength of comparative efficacy conclusions. Second, the study population included clinically heterogeneous LRTI subtypes with potentially varying pathophysiology, severity, and expected responses to antibiotic therapy. Crucially, the study lacked comprehensive microbiological data. The absence of detailed reporting on pathogen distribution, culture positivity rates, and antimicrobial susceptibility profiles means that the observed outcomes cannot be reliably attributed to the antimicrobial activity of the regimens against specific causative organisms. This fundamental gap severely weakens any causal interpretation regarding antimicrobial effectiveness and limits the clinical applicability of the findings to empiric therapy settings without microbiological guidance. Third, the inherent pharmacological differences between piperacillin-tazobactam and cefotaxime-tazobactam (spectrum, tissue penetration, PK/PD profiles) may confound direct efficacy comparisons, despite our efforts to align dosing frequencies. Fourth, the single-center design and moderate sample size may limit generalizability and statistical power for safety endpoints. Given these fundamental design limitations, the findings should be interpreted as associative and hypothesis-generating rather than confirmatory. Future large-scale, multicenter, randomized controlled trials with microbiological stratification are warranted to validate these findings.

Collectively, the results of this study indicate that both antibiotic regimens are viable therapeutic options for elderly patients with LRTIs in our setting. The observation of numerical differences favoring the PIP-TAZ group across multiple parameters generates the hypothesis that piperacillin-tazobactam might offer advantages in certain clinical scenarios-a hypothesis that requires rigorous testing in future studies. However, as emphasized throughout this manuscript, these numerical differences do not imply clinical superiority and must be interpreted with extreme caution due to the study’s non-randomized design and other inherent limitations. However, it is imperative to reiterate that the observational nature of this study, coupled with its methodological constraints, means that these associations cannot be construed as evidence of causal superiority. The primary contribution of this work lies in providing preliminary comparative data and underscoring the importance of designing more rigorous, randomized studies to guide antibiotic selection in the elderly.

## 6 Conclusion

In this prospective, non-randomized comparative study, both piperacillin-tazobactam and cefotaxime-tazobactam were associated with clinical and physiological improvements in elderly patients with LRTIs. Observed outcomes numerically favored piperacillin-tazobactam regarding improvements in oxygenation, inflammatory marker reduction, and lung function recovery, with a comparable safety profile to cefotaxime-tazobactam. However, due to the study’s non-randomized design, heterogeneous population, lack of comprehensive microbiological data, and exploratory statistical approach, these findings cannot establish the superiority of one regimen over the other and should be interpreted strictly as hypothesis-generating for future randomized controlled trials.

## Data Availability

The original contributions presented in the study are included in the article/supplementary material, further inquiries can be directed to the corresponding author.

## References

[B2] Chai K. M. Wang H. Guan Y. Shi R. Stalin A. Zhai Y. (2024). Effectiveness of bairui granules in the treatment of respiratory tract infections: a systematic review and meta-analysis. Medicine (Baltimore). 103 (29), e38904. 10.1097/MD.0000000000038904 39029033 PMC11398807

[B4] Chen C. Deng L. Liu S. Q. Du Q. Xi X. (2023). Analysis of reports of drug-induced anaphylactic shock in chongqing from 2015 to 2020. Chin. J. Pharmacovigil. 20 (4), 460–464. 10.19803/j.1672-8629.20220570

[B5] Expert Committee of Chinese Laboratory Medicine Training Project, Chinese Society of Respiratory Medicine (2023). Expert consensus on clinical application of nucleic acid detection technology for pathogen diagnosis of respiratory tract infections in adults (2023). J. Peking Union Med. Coll. 14 (5), 959–971. 10.12290/xhyxzz.2023-0338

[B6] Grau S. Lozano V. Valladares A. Cavanillas R. Xie Y. Nocea G. (2014). Antibiotic expected effectiveness and cost under real life microbiology: evaluation of ertapenem and ceftriaxone in the treatment of community-acquired pneumonia for elderly patients in Spain. Clin. Outcomes Res. 6, 83–92. 10.2147/CEOR.S55265 24611019 PMC3928454

[B1] Ji B. Li S. R. Guan Y. Cao S. S. Chen B. F. Zhang W. (2024). Efficacy and safety of cefoperazone-sulbactam and piperacillin-tazobactam in treatment of complicated urinary tract infection. Chin. J. Nosocomiol. 34 (6), 841–846. 10.11816/cn.ni.2024-231044

[B7] Liu X. H. Xu B. P. Shang Y. X. Zhang H. Zhang Z. K. Lin G.Y. (2025a). Efficacy and safety of aerosol inhaled recombinant human interferon α1b in the treatment of respiratory syncytial virus lower respiratory tract infection in children: a multicenter, randomized, double-blind, placebo-controlled phase III clinical study. Chin. J. Pract. Pediatr. 40 (3), 180–186. 10.3760/cma.j.cn101070-20241028-00694

[B8] Liu T. Zhang T. Y. Ma L. Zhao Q. R. Zhang J. H. Wang Y. (2025b). Research progress on immune regulation and clinical treatment strategies in respiratory virus infection. Chin. J. Immunol. 41 (1), 231–240. 10.3969/j.issn.1000-484X.2025.01.036

[B9] Mauritz M. D. Von Both U. Dohna-Schwake C. Gille C. Hasan C. Huebner J. (2024). Clinical recommendations for the inpatient management of lower respiratory tract infections in children and adolescents with severe neurological impairment in Germany. Eur. J. Pediatr. 183 (3), 987–999. 10.1007/s00431-023-05401-6 38172444 PMC10951000

[B10] Metlay J. P. Waterer G. W. Long A. C. Anzueto A. Brozek J. Crothers K. (2019). Diagnosis and treatment of adults with community-acquired pneumonia. An official clinical practice guideline of the American thoracic society and infectious diseases society of America. Am. J. Respir. Crit. Care Med. 200 (7), e45–e67. 10.1164/rccm.201908-1581ST 31573350 PMC6812437

[B11] Milani G. P. Alberti I. Abodi M. Lakoumentas J. Konstantinou G. N. Papadopoulos N. G. (2024). A systematic review and meta-analysis on nutritional and dietary interventions for the treatment of acute respiratory infection in pediatric patients: an EAACI taskforce. Allergy 79 (7), 1687–1707. 10.1111/all.15997 38174413

[B14] Shi Y. (2024). Pay attention to the clinical rational application of polymyxin aerosol inhalation in the treatment of multidrug-resistant Gram-negative lower respiratory tract infections. Chin. J. Clin. Infect. Dis. 17 (5), 371–374. 10.3760/cma.j.issn.1674-2397.2024.05.005

[B12] Wang X. (2023). Pediatric branch of the Chinese association of traditional Chinese medicine. Expert consensus on the clinical application of xiaoer resuqing oral liquid in the treatment of upper respiratory tract infections in children. Chin. J. Traditional Chin. Med. 41 (12), 253–258. 10.13193/j.issn.1673-7717.2023.12.048

[B13] Wang M. L. Ju Y. H. Wu R. Zhu G. C. Xu X. J. Zhu Y. (2023). Physical compatibility of amifostine with 17 intravenous drugs in a simulated Y-type infusion pathway. Pract. Drugs Clin. 26 (5), 448–452. 10.14053/j.cnki.ppcr.202305012

[B15] Wang Y. Zhou Y. (2024). Study on the efficacy of piperacillin -tazobactam sodium and cefoperazone -sulbactam sodium in the treatment of severe pneumonia in the elderly. J. North Sichuan Med. Coll. 39 (6), 843–846. 10.3969/j.issn.1005-3697.2024.06.030

[B16] Xiao Y. Liu M. R. (2025). Distribution of pathogens in hospital bloodstream infection and the value of MALDI-TOFMS in rapid identification of pathogens. Chin. J. Pathogenic Biol. 20 (1), 71–76. 10.13350/j.cjpb.250113

[B17] Zhang X. X. Zhang C. L. Ren T. Wang J. Cheng J. (2024). Pathogen characteristics and drug resistance status of patients in respiratory medicine. South China J. Prev. Med. 50 (5), 478–481. 10.12183/j.scjpm.2024.05097

[B3] Zhao C. J. Ou Y. B. (2024). Clinical study on baoerning oral liquid combined with auricular acupoint therapy for the treatment of lung and spleen qi deficiency syndrome in children with recurrent respiratory tract infection. China Med. Her. 21 (14), 83–86. 10.20047/j.issn1673-7210.2024.14.22

